# From Tick Bite to Broken Heart: A Case of Stress Cardiomyopathy Induced by Severe Babesiosis

**DOI:** 10.7759/cureus.84612

**Published:** 2025-05-22

**Authors:** Marie Yung-Chen Wu, Sokratis N Zisis, Thomas C Tsai, Palapun Waitayangkoon, Felipe Barbosa

**Affiliations:** 1 Department of Internal Medicine, MetroWest Medical Center, Framingham, USA; 2 Department of Medicine, Tufts University School of Medicine, Boston, USA; 3 Department of Medicine, MetroWest Medical Center, Tufts University School of Medicine, Framingham, USA; 4 Department of Infectious Diseases, MetroWest Medical Center, Tufts University School of Medicine, Framingham, USA

**Keywords:** acute hypoxemic respiratory failure, severe babesiosis, takosubo cardiomyopathy, tick-borne infections, zoonosis and public health

## Abstract

Babesiosis can present with a wide range of clinical manifestations, from asymptomatic infection to severe, life-threatening illness. We report the case of a 74-year-old woman with severe babesiosis complicated by Takotsubo cardiomyopathy. She presented with fever, chills, fatigue, and shortness of breath two weeks after a tick bite of unknown attachment duration. Initial testing for tick-borne illnesses, including *Borrelia*, *Ehrlichia*, *Anaplasma*, and *Babesia*, was negative. As her condition progressed, she developed hemolytic anemia, and a repeat peripheral blood smear revealed 4.5% parasitemia, confirming severe babesiosis. Despite treatment with atovaquone and azithromycin, her clinical status deteriorated, necessitating exchange transfusion, mechanical ventilation for respiratory failure, and hemodynamic support with inotropes and vasopressors for refractory cardiogenic shock. Echocardiography confirmed Takotsubo cardiomyopathy, which resolved with appropriate treatment. This case underscores the diagnostic complexity and potential severity of babesiosis, highlighting the importance of maintaining a high index of suspicion and initiating prompt therapy to prevent serious complications.

## Introduction

Babesiosis is a tick-borne zoonotic infection caused by *Babesia *species, primarily *Babesia microti*, in the United States. In North America, transmission typically occurs through the bite of an infected deer tick (*Ixodes *species) during its blood meal, with the disease most prevalent in the Northeast and Midwest. Globally, babesiosis has been reported on nearly every continent, with most human cases occurring in Europe and parts of Asia. Other modes of transmission include blood transfusions, organ transplantation, and, in rare cases, perinatal transmission from mother to child [1]. The parasite’s life cycle alternates between a mammalian host, where it reproduces asexually within red blood cells, and a tick vector, where sexual reproduction and sporogony take place [1]. The incidence of babesiosis has increased over time, with the number of cases reported annually to the CDC rising from 1,126 in 2011 to 2,418 in 2019 [2]. Clinically, babesiosis presents along a broad spectrum, ranging from asymptomatic infection to severe, potentially fatal disease, particularly among immunocompromised individuals, such as the elderly and those without a spleen [3].

This case report aims to provide an overview of the current understanding of babesiosis, with a focus on its epidemiology, clinical features, diagnostic methods, treatment strategies, and key insights gained from the present case.

This article was previously presented as a meeting abstract at the 2024 CHEST Annual Meeting on October 7, 2024 [4].

## Case presentation

A 74-year-old woman with asthma and a history of giant cell arteritis presented to the emergency room with fever, chills, fatigue, exertional dyspnea, and a nonproductive cough lasting one week. On admission, she was febrile with a temperature of 39.4°C, tachycardic with a heart rate of 124 beats per minute, hypertensive with a blood pressure of 151/67 mmHg, tachypneic with a respiratory rate of 22 breaths per minute, and had an oxygen saturation of 94% on room air. She reported using her albuterol inhaler as needed at least four times daily. She denied chest pain, palpitations, dysuria, changes in bowel movements, or flank pain. Two weeks before the presentation, she had sustained a tick bite while vacationing in the northeastern United States. The removed tick was not engorged, and although the duration of attachment was unknown, no subsequent rash developed. She was a former smoker and did not use alcohol or substances other than marijuana. Her family history was significant for liver failure in her father and pancreatic cancer in her mother.

On physical examination, she was febrile, tachycardic, and tachypneic. Auscultation revealed bilateral diffuse rhonchi. Her skin was warm and intact, without any bite lesions or rash. The rest of the examination was unremarkable. Initial laboratory tests showed leukocytosis (16.5 K/μL) with a left shift, normal hemoglobin, and a normal platelet count. Renal function was within normal limits, while liver transaminases were elevated (alanine transferase: 70 U/L; aspartate transaminase: 84 U/L), along with an increased total bilirubin (1.3 mg/dL). PCR tests for SARS-CoV-2 and influenza A and B were negative.

An elevated D-dimer level (4.85 µg/mL) prompted evaluation for thromboembolism, which was excluded by CT angiography of the chest. The CT also showed no evidence of consolidation or pleural effusions. Testing for tick-borne diseases - including peripheral smears for *Ehrlichia*, *Anaplasma*, and *Babesia*; Lyme antibodies; and blood cultures - was initially negative. Empiric doxycycline was started to cover potential tick-borne illnesses and atypical respiratory infections. Despite this, the patient’s condition worsened, with persistent fevers and worsening shortness of breath. Hemoglobin and platelet counts declined. Persistently elevated liver enzymes led to an abdominal ultrasound, which revealed nodularity of the liver capsule but no abnormalities in the gallbladder or biliary tract. A CT scan of the abdomen and pelvis confirmed nodular changes in the liver contour, while magnetic resonance cholangiopancreatography ruled out biliary duct dilation. Additional testing for HIV, Epstein-Barr virus, cytomegalovirus, fungi, and hepatitis viruses was negative. Doxycycline was discontinued, and empiric broad-spectrum antibiotics with piperacillin/tazobactam were initiated.

Despite treatment, the patient remained febrile and developed progressive anemia. A hemolysis panel revealed an elevated lactate dehydrogenase (LDH) level of 658 U/L. A repeat set of thin and thick blood smears for parasites showed 4.5% *Babesia *parasitemia. Atovaquone and azithromycin were started, and piperacillin/tazobactam was discontinued. However, her hemolytic anemia worsened, with hemoglobin dropping to 8 g/dL, prompting the need for exchange transfusion. Table 1 summarizes her laboratory values from admission to the day of exchange transfusion.

**Table 1:** Laboratory values from Day 1 to the day of exchange transfusion The patient’s leukocytosis persisted throughout this period, while HGB and HCT levels progressively declined. Thrombocytopenia worsened, and bilirubin levels showed a continued upward trend. HCT, hematocrit; HGB, hemoglobin

Day	WBC (10³/μL; reference range: 4.0-11.0)	HGB (g/dL; reference range: 12.0-16.0)	HCT (%; reference range: 36.0-48.0)	Platelet (10³/μL; reference range: 150-400)	Total bilirubin (mg/dL; reference range: 0.2-1.0)
1	16.5	12.3	36.8	12.3	1.3
2	13.8	11.3	33.2	11.3	1.1
3	11.4	11.3	33.9	11.3	1.3
4	14.5	11.9	35.7	11.9	2.6
5	14.8	10.1	31.4	10.1	3
6	15.3	9.2	28	9.2	3.5
7	14.9	8	25.2	8	2.3

**Figure 1:**
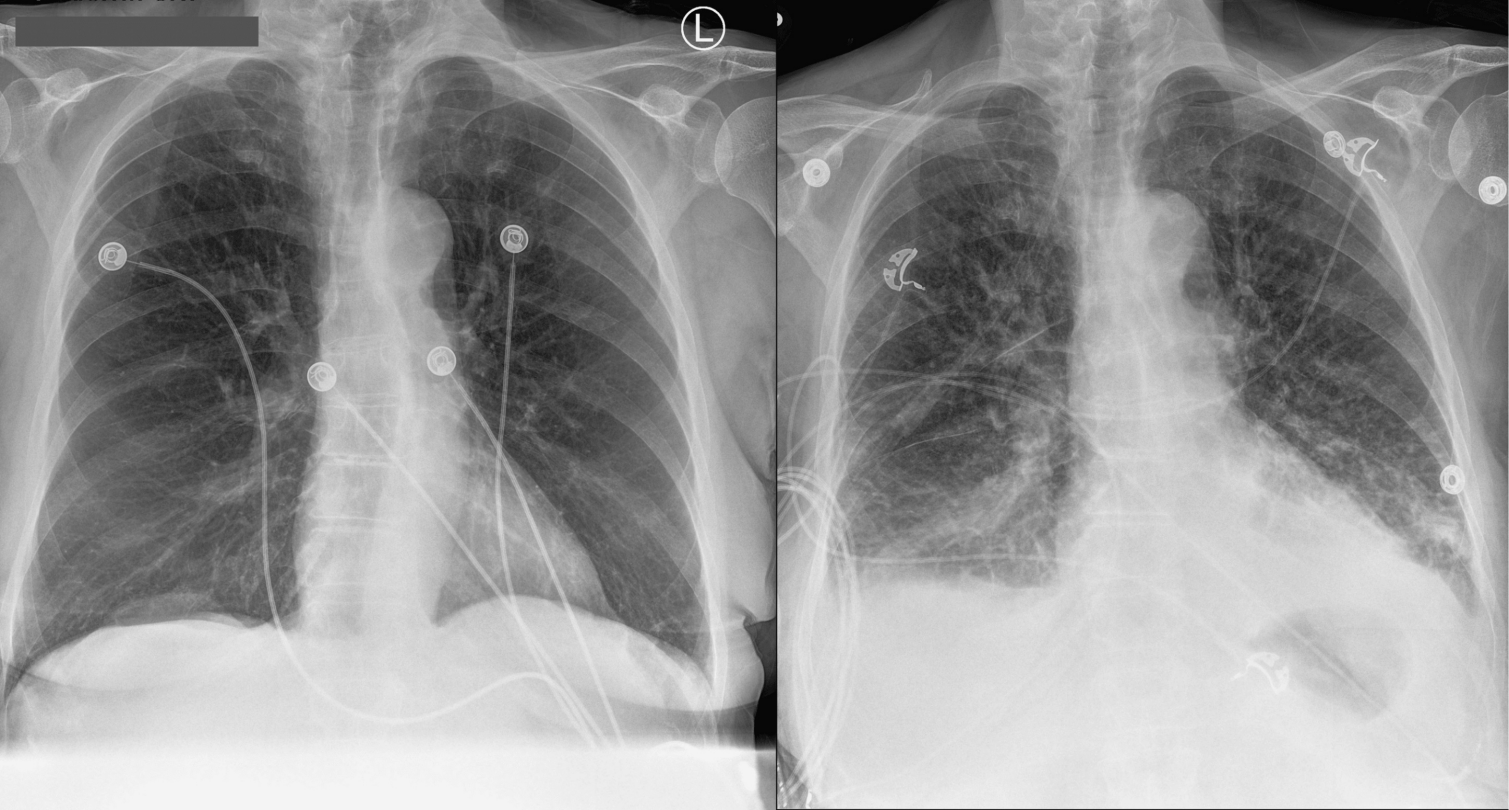
Chest X-rays on the day of admission (left) and on the day of exchange transfusion (right) for comparison The admission chest X-ray (left) shows clear lung fields with no evidence of pulmonary edema. In contrast, the chest X-ray obtained on the day of exchange transfusion (right) reveals newly developed pulmonary edema.

**Figure 2:**
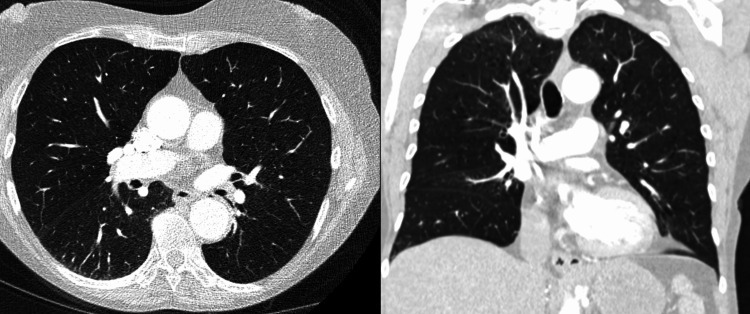
Interim CT scan of the chest The interim chest CT scan shows no evidence of pulmonary edema or pleural effusions, indicating that pulmonary edema developed later in the clinical course. Left: axial view; right: coronal view.

During the exchange transfusion, the patient’s dyspnea and oxygen desaturation worsened, requiring 4 L/min of oxygen via nasal cannula. Shortly after completing the procedure, she needed supplemental oxygen delivered by a non-rebreather mask at maximum flow rate. Laboratory tests showed a markedly elevated pro-B-type natriuretic peptide level of 15,716 ng/mL. Chest X-ray revealed cardiomegaly, engorged pulmonary vasculature, and bilateral pleural effusions, consistent with acutely decompensated heart failure. Bedside echocardiography demonstrated global hypokinesis with a left ventricular ejection fraction estimated between 15% and 20%. Despite prompt diuresis, her respiratory distress progressed, necessitating intubation and mechanical ventilation. She subsequently developed refractory cardiogenic shock, requiring vasopressor support with norepinephrine and inotropic support with dobutamine. Fortunately, parasitemia responded well to atovaquone and azithromycin, achieving complete clearance after five days. Antimicrobial therapy was continued for an additional 14 days. Follow-up echocardiography showed normalization of ejection fraction, confirming cardiomyopathy of stress-induced etiology. Figure 1 compares chest X-rays from the day of admission (left) and the day of exchange transfusion (right). Figure 2 shows the interim chest CT scan demonstrating the absence of pulmonary edema or pleural effusions (left: axial view; right: coronal view).

## Discussion

Human babesiosis, caused by the *Babesia *protozoan, became a nationally notifiable disease in the United States in January 2011 [5]. In the United States, tick-borne transmission occurs most frequently in the Northeast and upper Midwest, with cases peaking during the warmer months. The clinical presentation varies widely, ranging from mild illness to severe, life-threatening disease [6,7].

A case series of 34 patients with severe babesiosis reported an average time to diagnosis of 15.4 days (range: three to 44 days) and an average hospital stay of 13 days [3]. Hospitalized patients often have underlying comorbidities such as cancer, HIV infection, hemoglobinopathies, or chronic cardiac, pulmonary, or hepatic conditions. Splenectomy is another known risk factor. Diagnosis is frequently delayed due to nonspecific symptoms, including fever, chills, sweats, headache, anorexia, and myalgia [8].

Laboratory evaluation typically reveals anemia, often preceded by severe thrombocytopenia. Leukocytosis may also be present. Elevated LDH and bilirubin levels are indicative of hemolytic anemia [9]. A definitive diagnosis is usually made through identification of intraerythrocytic parasites on Giemsa-stained thin blood smears. First-line treatment for immunocompetent individuals includes atovaquone and azithromycin or clindamycin and quinine, typically administered for seven to 10 days [1]. In patients with severe disease and high parasitemia, red blood cell exchange transfusion is often used alongside antimicrobials to reduce the parasite load and improve clinical outcomes [8,10].

In this case, there was high clinical suspicion for tick-borne illnesses, including Lyme disease (*Borrelia burgdorferi*), babesiosis, ehrlichiosis, and anaplasmosis. As a result, empiric antimicrobial therapy was initiated prior to a definitive diagnosis. However, initial thick and thin blood smears were negative for tick-borne pathogens. This prompted further evaluation due to persistent leukocytosis and abnormal cholestatic liver function tests. In hindsight, early infection with a low parasite burden and variability between operators likely contributed to the false-negative smear results. Literature supports repeating blood smears every 12 to 24 hours or using *Babesia*-specific PCR when clinical suspicion remains high [7,8].

Ultimately, the patient was diagnosed with severe babesiosis despite lacking apparent risk factors at presentation. Retrospective imaging, however, revealed undiagnosed cirrhosis, which may have contributed to disease severity. She was treated with atovaquone and azithromycin. Due to worsening respiratory symptoms, hemolytic anemia, thrombocytopenia, and 4.5% parasitemia, exchange transfusion was performed as an adjunct to antimicrobial therapy. Her respiratory condition deteriorated during and after the transfusion, necessitating mechanical ventilation in the ICU. Echocardiography confirmed stress-induced (Takotsubo) cardiomyopathy as a complication.

While severe babesiosis is known to cause pulmonary edema, acute respiratory distress syndrome, and congestive heart failure [3,5], stress-induced cardiomyopathy is rarely reported, with only two documented cases in the literature [11]. Further research is needed to better understand the pathophysiology and potential link between babesiosis and stress-induced cardiomyopathy.

Severe babesiosis carries a significant risk of mortality. Among hospitalized patients, earlier case series reported mortality rates ranging from 6.5% to 8.8% [3,12]. Additionally, a 30-day mortality rate of 3.3% was observed in Medicare beneficiaries diagnosed with babesiosis between 2006 and 2013 [13].

## Conclusions

Our case illustrates shortness of breath resulting from stress-induced cardiomyopathy triggered by severe babesiosis, an uncommon and diagnostically challenging presentation. The initial differential diagnosis included several tick-borne illnesses, such as Lyme disease, babesiosis, ehrlichiosis, and anaplasmosis. However, early thick and thin blood smear microscopy for tick-borne pathogens was negative. When clinical suspicion for babesiosis remains high despite negative initial smears, repeat blood smear examinations are crucial. This diagnostic challenge highlights the complexity of babesiosis and the potential for delayed diagnosis, particularly given its nonspecific early symptoms.

Stress-induced cardiomyopathy as a complication of babesiosis is extremely rare. This case adds to the limited literature on atypical cardiac involvement associated with the disease. While antimicrobial therapy helps reduce parasitemia, exchange transfusion may be required in severe cases to prevent rapid clinical decline. Regular monitoring of parasite load via blood smears is essential to guide the duration and effectiveness of treatment. Thus, a thorough understanding of babesiosis and its diverse clinical manifestations is key to improving diagnostic accuracy and optimizing patient outcomes. Clinicians should remain alert to rare presentations like this one to enable timely recognition and appropriate management of this potentially life-threatening infection.
